# Acquired Factor VIII Deficiency in Chronic Myeloid Leukemia: A Case Report

**DOI:** 10.7759/cureus.48802

**Published:** 2023-11-14

**Authors:** Waheed Ul Hassan, Muhammad Ahmad, Basel Abdelazeem, Ujwala Koduru, Arvind Kunadi

**Affiliations:** 1 Internal Medicine, Mclaren Flint Hospital, Flint, USA

**Keywords:** factor deficiency, case report, acquired hemophilia, chronic myeloid leukemia, factor viii

## Abstract

Factor VIII deficiency is mostly seen in hemophilia A, an X-linked recessive disorder. Patients would have a past medical history of bleeding diathesis or a family history of bleeding disorder. Acquired deficiencies of factor VIII are rare; some cases have been reported in solid malignancies. We present this case of acquired factor VIII deficiency in chronic myeloid leukemia (CML). A 72-year-old man was incidentally found to have leukocytosis at 31,000 and a platelet count of 3.2 million on a routine complete blood count (CBC). Prothrombin time (PT), international normalized ratio (INR), and partial thromboplastin time (PTT) showed an isolated elevation of PTT at 38.1 and a low factor VIII activity level at 39. The patient did not have any history or physical examination suggestive of bleeding diathesis. A bone marrow biopsy confirmed the BCR/ABL mutation, a diagnosis of CML was made, and the patient was started on dasatinib for one month. His PTT normalized after treatment for CML, suggesting a deficiency of factor VIII likely related to CML. The aim of this study is to highlight a case with acquired factor VIII deficiency due to CML and to emphasize the importance of coagulation workup in all newly diagnosed CML patients.

## Introduction

Factor VIII deficiency can be inherited or acquired. Although inherited factor deficiency, also known as hemophilia A, is a relatively common entity, it is X-linked recessive and is almost exclusively seen in male children. Acquired factor VIII deficiency is relatively rare. It can be caused by antibody formation against factor VIII; for example, in systemic lupus erythematosus (SLE), antibodies not only against factor VIII but also against other factors, including IX, XI, XII, and II, are seen. The mechanism of autoantibody production is unclear. Although rare, acquired factor VIII deficiency has been reported in some solid organ malignancies [1]. Some drugs, such as penicillin, sulfonamides, and phenytoin, have been associated with the production of autoantibodies against factor VIII [2]. Some studies also report acquired factor VIII deficiency in pregnancy. We present a case of acquired factor VIII deficiency in a CML patient, which improved after treatment with a tyrosine kinase inhibitor.

Chronic myeloid leukemia (CML) is associated with changes in the hemostatic system and may result in thrombotic or hemorrhagic complications [3]. One of the derangements is acquired factor VIII deficiency, which significantly improved after treatment with a tyrosine kinase inhibitor, i.e., dasatinib. There are very few case reports in the literature suggesting acquired factor VIII deficiency in CML [4]. This case report presents a patient who was diagnosed with CML after a routine complete blood count (CBC) showed marked leukocytosis and thrombocytosis. The patient was also found to have an isolated partial thromboplastin time (PTT) elevation and a low factor VIII activity level, which normalized after one month of treatment with dasatinib. The aim of this study is to highlight the importance of coagulation studies in CML and consider the possibility of factor deficiencies in patients with isolated PTT elevation. There are studies that have demonstrated the derangement of various components of the hemostatic system resulting in thrombo-hemorrhagic complications in CML; however, there is only one case report in the literature that highlights acquired factor VIII deficiency in a patient with CML who was treated with interferon-alpha. We consider it important to perform coagulation studies and look for any potential coagulation issues in newly diagnosed CML patients.

## Case presentation

We present the case of a 72-year-old man with a significant past medical history for a left-sided cerebrovascular accident about 15 years ago, hypothyroidism, hypertension, and dyslipidemia. His social history was significant for smoking one pack in three to four days since the age of 20 years. He reported exposure to Agent Orange in the Vietnam War, lived alone, walked with a cane due to residual deficits from the past stroke, and denied any drinking or illicit drug use. According to the patient, there is no family history of cancer, heart disease, diabetes, or stroke. 

The patient visited the primary care office for a regular follow-up on his medical conditions. The patient denied any active complaints, including any new or progressive B symptoms, significant weight loss, fever or recurrent infections, or drenching sweats. The physical examination was negative for any palpable lymphadenopathy or organomegaly. His medical conditions were stable, and he reported to be compliant with home medications, i.e., Lisinopril 5 mg daily, Atorvastatin 40 mg daily, Synthroid 125 mcg daily, and Plavix 75 mg daily. 

Routine blood work, including CBC, comprehensive metabolic panel (CMP), and lipid profile, was ordered. CBC showed a WBC count of 31,000 and a platelet count of 3.2 million. Blood cultures came back negative for any growth. Hematology and oncology were consulted, and the patient was admitted for a bone marrow biopsy (Table 1).

**Table 1:** CBC at the time of diagnosis and compares it with repeat CBC one month after treatment with dasatinib. The WBC count improved, while the platelet count decreased. CBC: complete blood count; MCV: mean corpuscular volume; MCH: mean corpuscular hemoglobin.

CBC	May 19, 2022	June 28, 2022	Reference range
WBC	31.01x10^3^/uL	7.6x10^3^/uL	4.5-11.0x10^3^/uL
Platelets	3258x10^3^/uL	1541x10^3^/uL	140-440x10^3^/uL
Hemoglobin	13.1 g/dL	12.2 g/dL	13.5-7.7 g/dL
RBC	4.41x10^6^/uL	4.43x10^6^/uL	4.70-6.10x10^6^/uL
MCV	96.9 fL	97 fL	80-94 fL
MCH	29.6 pg	32.2 pg	27-31 pg

Coagulation profiles, including prothrombin time (PT)/international normalized ratio (INR) and PTT, done at the time of admission for bone marrow biopsy showed an isolated elevation of PTT at 38.1 with correction to 29 on the 1:1 mixing study, and dilute Russell viper venom time (DRVVT) elevated at 55.6 with correction to 39.2 on the 1:1 mix, suggesting the possibility of coagulation factor deficiency. 

Factor VIII assay was done, which was low at 39. Lupus anticoagulant (LA) ratio and LA ratio mix were within normal range at 1.07 and 1.04, respectively. No lupus inhibitor was recognized (Table 2).

**Table 2:** Coagulation and thrombosis profile of our patient. PTT improved to 32 after one month of treatment with dasatinib. DRVVT: dilute Russell viper venom time; PTT: partial thromboplastin time; PT: prothrombin time; INR: international normalized ratio; LA: lupus anticoagulant.

Coagulation/thrombosis	May 22, 2022	June 28, 2022	Reference range
Factor VIII assay	39%		49-71%
DRVV	55.6 seconds		31-44 seconds
DRVVT 1:1 mix	39.2 seconds		31-44 seconds
PTT	39.8 seconds	32 seconds	24.7-29.9 seconds
PTT 1:1 mix	29 seconds		24.7-29.9 seconds
PT	13.4 seconds	11.6 seconds	9.5-12.0 seconds
INR	1.25		0.88-1.11
LA ratio	1.07		
LA ratio mix	1.04		

Bone marrow biopsy showed hypercellularity, with trilineage hematopoiesis. Myeloid hyperplasia was noted with an increase in myelocytes, eosinophils, and basophils. Flow cytometry did not show any monotypic B-cell population or increase in blasts. The BCR/ABL was detected at a level >50,000. JAK mutation was negative. The patient was started on dasatinib. Repeat PTT after one month of treatment was normalized, and leukocytosis improved to the normal range. Thrombocytosis improved significantly with platelet reduction to 50% of the initial number.

The patient did not report any bleeding problems, such as mucosal bleeding, gastrointestinal (GI) bleeding, and ecchymosis, before or after one month of treatment.

## Discussion

CML is a disorder characterized by the proliferation of myeloid stem cells. The reason for this overproliferation is BCR-ABL chimeric gene product, which is a constitutively active tyrosine kinase. The increased activity of tyrosine kinase leads to excessive replication of a mutated clone of myeloid stem cells, and over time, this leads to suppression of otherwise normal hematopoiesis [5]. Tyrosine kinase inhibitors, such as dasatinib, can suppress these mutated clones, and normal hematopoietic cells can restore normal hematopoiesis [6].

Most patients presenting to the clinic are asymptomatic because they are in the indolent or chronic phase of CML. Patients may complain of abdominal fullness due to splenomegaly, fatigue, or malaise. 

Symptoms of bleeding diathesis or thrombosis-related complications are infrequent initial presentations of CML, and this is because several components of the hemostatic system are reported to be deranged. Leukostasis is one of the causes of these derangements. Drugs used to treat leukostasis have also been associated with hemostatic issues. Acquired factor deficiency, especially factor VIII, also appears to be present in some cases of CML [7]. English et al. reported a case of acquired factor VIII inhibitor in a patient with CML receiving interferon therapy [8]. Autoantibodies develop against factor VIII, leading to acquired hemophilia A, and this has been seen in postpartum period, rheumatoid arthritis, malignancy, SLE, and drug reactions, and even without any underlying disease in as many as 50% of cases [9].

Acquired factor VIII deficiency is caused by autoantibody formation against factor VIII. It is less common as compared to congenital deficiency of factor VIII [10]. Acquired deficiency of factor VIII is seen in malignancies, pregnancy, and SLE and has also been reported to be associated with some drugs like phenytoin and sulfonamides [11]. The exact mechanism of the development of autoantibodies is not clear, so the pathogenesis of acquired factor VIII deficiency is not clear. CML has also been associated with several hematologic derangements. Factor VIII deficiency, which appears to be acquired, has been reported in CML after treatment with interferon alpha. Our case highlights factor VIII deficiency in a newly diagnosed CML patient [12].

Factor VIII, which is an essential factor for the intrinsic pathway of the coagulation cascade, can be deficient congenitally due to a mutation of the *F8* gene on the X chromosome, leading to hemophilia A [13]. Another common cause of low factor VIII level is von Willebrand disease (vWD), the reason being that von Willebrand factor is needed to bind to factor VIII and store it in the subendothelium. However, these disorders have been frequently present since childhood and have a positive family history [14].

This case describes an acquired deficiency of factor VIII, which we consider most likely to be secondary to CML. The patient had isolated PTT elevation and low factor VIII activity level, which normalized after treatment with a second-generation tyrosine kinase inhibitor, dasatinib. The patient did not report any bleeding diathesis, such as mucosal bleeding, nosebleed, ecchymosis, or GI bleeding. 

Although infrequent, isolated elevation of PTT in the older population is likely acquired, and etiologies such as malignancies and autoimmune processes should be considered. Isolated elevation of the activated PTT (aPTT) can be used as an initial screening test, which can be followed by a mixing study or inhibitor screen [9,10]. Correction with mixing can confirm the factor deficiency; otherwise, further testing to find inhibitors of factor VIII can include the addition of phospholipid and Bethesda assay [15].

This case report opens prospects to explore coagulation problems in CML. We have presented a case of factor VIII deficiency in CML; however, more cases are needed to highlight coagulation problems, such as other acquired factor deficiencies, which need to be explored more in CML. The exact mechanism and pathogenesis of factor VIII deficiency are not understood. Further studies are needed to determine the magnitude and pathogenesis of factor VIII deficiency in CML. 

## Conclusions

Acquired factor VIII deficiency has been seen in some solid malignancies, especially adenocarcinomas and hematological malignancies, as well as in autoimmune conditions, such as rheumatoid arthritis, SLE, and the postpartum period. This case highlights a secondary factor VIII deficiency in a patient with newly diagnosed CML, which was not significant to cause any symptoms in the patient. Although clinically nonsignificant, clinician should be alerted to prolonged aPTT, which can be an acquired factor VIII deficiency in CML patients.
